# Physical Activity, Readiness, and Cardiovascular Risk Stratification in the Polytechnics Communities of the Northern Region of Portugal Integrated in Mobility as a Service Concept

**DOI:** 10.3390/healthcare11243145

**Published:** 2023-12-11

**Authors:** Andreia S. P. Sousa, Diana C. Guedes, José Félix, Soraia Pereira, Rubim Santos

**Affiliations:** Center for Rehabilitation Research (CIR), ESS, Polytechnic of Porto, Rua Dr. António Bernardino de Almeida, 400, 4200-072 Porto, Portugal; dcrg@ess.ipp.pt (D.C.G.); josefelixfelix15@gmail.com (J.F.); sfap@ess.ipp.pt (S.P.); rss@ess.ipp.pt (R.S.)

**Keywords:** physical activity, readiness, university

## Abstract

The aim of the study is to characterize physical activity (PA) levels and PA readiness as well as stratify cardiovascular risk among the population of polytechnics community members in the north region of Portugal, including students, academic teachers, and non-teacher staff. An online questionnaire about general sample characterization, PA level, and readiness was applied. Of the 717 respondents, 237 were academic teachers, 143 were non-teacher staff, and 337 were students. Most of the participants had a level of moderate PA, including students, academic teachers, and non-teacher staff (82%). The sedentary behavior was higher in the academic teachers and non-teacher staff groups. A total of 56% of the participants had low cardiovascular risk; the group of students were the population with higher risk. Approximately half of the participants need to consult a qualified professional before increasing their PA. Overall, the participants presented moderate levels of PA, although there is still a considerable number of sedentary people that must be considered.

## 1. Introduction

Physical activity (PA) has been defined by the World Health Organization (WHO) as any bodily movement produced by skeletal muscles that requires energy expenditure. This includes all movement performed during leisure time, for transport to get to and from places, or as part of a person’s job [1]. According to the American College of Sports Medicine (ACSM), an adult should do at least 150–300 min of moderate-intensity aerobic PA or at least 75–150 min of vigorous-intensity aerobic PA per week [2]. These habits have some benefits, such as: improving muscular and cardiorespiratory fitness and bone and functional health; reducing the risk of several diseases, such as heart disease, stroke, hypertension, diabetes, several cancers, depression, and the risk of falls; and helping to maintain healthy body weight, improve mental health, quality of life, and well-being [1,3,4,5,6,7,8,9,10].

Despite the previously mentioned recommendations, and according to the Eurobarometer made in May of 2022, it is estimated that 62% of people in the European Union do not do any type of exercise and 50% are sedentary. In Portugal, the numbers are even scarier, since it is estimated that 78% of people do not do any physical training and 83% are sedentary [11]. Physical inactivity has been stated as the 4th leading risk factor for mortality [1], therefore, actions promoting an increase in PA are required to reduce the negative impact on health systems, the environment, economic development, community well-being, and quality of life [12].

Compliance with the recommendations for the level of PA during mobility may be a strategy to adopt, by promoting walking and cycling as the key means of transportation. This strategy has the potential to increase physical conditioning for all people, of all ages, and is consistent with valuing health as a universal right and an essential resource for everyday living and not just the absence of disease or infirmity [12]. Therefore, the connection of the concept of PA with the concept of Mobility as a Service (MaaS) would be a strategy to attain this objective [13,14] by including moments of PA in a distribution model that delivers users’ transport needs through one single interface of a service provider, combining different transport modes to offer tailored mobility packages [13]. Moreover, MaaS, by being a promotor of a cheaper and sustainable means of transport, has effects on the environment and the economy [15].

Considering the MaaS concept, mobility is a part of the construction of green campuses in response to the need to promote sustainability among members of the university community [15,16]. University students spend most of their time in classes, studying, or in front of the computer, so there is a risk of adopting sedentary behavior [6]. The communities of academic teachers and non-teacher staff can also have the same risks of adopting sedentary behavior because of their work lifestyle and screen time [16]. Especially for the community of academic teachers, whose work is not limited to tasks related to teaching and research, including a high workload doing considerable administrative and management activities such as preparing classes, correcting assignments, and producing excessive documentation with limited motor functions can increase their levels of stress and indicate worse levels of well-being [8,17,18]. When attempting to determine the amount of PA in this population, only one study was able to provide information concerning a sedentary lifestyle pattern in students from a public university in the northern part of Portugal [5]. While the academic sports associations (semi-professional and amateur sports within the scope of activities at universities like the *Federação Académica do Desporto Universitário* (FADU) in Portugal) contribute to an increase in PA, it is only that subset of PA characterized as being recreational, governed by rules, and orientated towards performance. Therefore, other strategies integrated into everyday life are needed. Also, it appears that no studies have yet been carried out that involve additional university populations from these areas, such as academic teachers and non-teacher staff. Moreover, other information needs to be considered in the characterization of this population, such as the readiness for PA and the risk stratification as recommended by ACSM [2]. Indeed, the ACSM states that while it is not anticipated that PA would cause any kind of cardiovascular incident in healthy individuals, in conditions with cardiovascular disease or risk factors, there is an increased risk of sudden death and/or myocardial infarctions when performing vigorous exercise [2]. Therefore, a pre-participation screening algorithm should be applied, being essential to evaluate the level of cardiovascular risk and the PA readiness in order to conduct safe PA promotor programs [19,20].

Considering the abovementioned, the Technology, Environment, Creativity, and Health (TECH) project aims to develop a MaaS service that promotes sustainable moments of PA, favoring compliance with the WHO PA recommendations of reducing the level of sedentarism for the polytechnics community of Portugal’s north region.

To finetune the MaaS to the users’ needs and given the dearth of knowledge regarding the extent of PA among the university community, particularly among academic teachers and non-teacher staff, characterizing the levels of PA, readiness to perform PA, and cardiovascular risk is needed. Therefore, the purpose of this study is to characterize the levels of PA, readiness to perform PA, and cardiovascular risk of the polytechnics population of the of Portugal’s north region including students, academic teachers, and non-teacher staff.

## 2. Materials and Methods

### 2.1. Participants

A cross-sectional study was designed to characterize the levels of PA, readiness to perform PA, and cardiovascular risk by sampling two of the four polytechnic institutes of the north region of Portugal: the Polytechnic Institute of Porto (P. PORTO) and Polytechnic Institute of Viana do Castelo (IPVC). This study was performed under a more global research project aiming to develop technologies in the domain of the Mobility as a Service concept to promote health in the north region of Portugal’s polytechnic institutes by increasing the levels of PA. This characterizing data will be used to meet the real needs of our target population, which is the academic community. The sample included students (a person enrolled in a degree or non-degree course in an academic institution), academic teachers (a person that teaches in some academic institution) and non-teacher staff (an individual who is a part of the academic institution and has the responsibility of promoting the proper functioning of the institution like administrative personnel, subordinates, cleaning, minders, and other jobs not involving teaching) fluent in Portuguese and residing in Portugal at the time of the study. Participants who presented one or more of the following criteria were excluded: (1) no access to the internet and/or (2) presents a health condition that prevents car sharing (mobility changes will not be considered).

The present study was submitted and approved by the Ethics Committee of the Health School of Polytechnic of Porto (CE0100B).

In accordance with the Helsinki Declaration, participants were fully informed of the study’s purpose before completing the questionnaire. They were only allowed to proceed after giving their informed consent in the questionnaire’s first part. No identifying information of the participants was collected.

### 2.2. Instruments and Procedures

Data was collected using an online questionnaire developed on the Google Forms platform and disseminated through institutional emails to students, academic teachers, and non-teacher staff from the P. PORTO and IPVC between June 2022 and October 2022. All the participants who completed the questionnaire and agreed to continue after giving informed consent were included in the study.

The questionnaire included questions for the following: sample characterization, PA level assessment, PA readiness assessment, and risk stratification for cardiovascular events according to the recommendations of the American College of Sports Medicine [2]. The whole questionnaire was previously tested in a pilot study involving 7 participants that were not included in the final sample.

#### 2.2.1. Characterization Data

For the sample characterization, a questionnaire was used to assess the following: age, weight, height, smoke habits, history of cardiovascular, respiratory, and/or metabolic disease, family history of coronary disease, diabetes or prediabetes, high blood pressure, cholesterol, and the presence of other health conditions.

#### 2.2.2. PA Level Assessment

The Portuguese version of the International Physical Activity Questionnaire—Short Form (IPAQ-SF) was used to measure the PA level. This instrument is composed of 7 questions structured to provide separate scores for activities such as walking, moderate-intensity activities, and vigorous-intensity activities in the last 7 days [21]. The metabolic equivalent task was calculated through the following formula [22]:

Total MET (minutes/week) = (Walk MET × minutes × days) + (Mod MET × minutes × days) + (Vig METS × minutes × days),(1)

where Total MET represents the total amount of energy expended throughout the whole week, Walk MET is the total amount of energy expended walking throughout a week, Mod MET is the total amount of energy expended doing moderate PA throughout a week, and Vig MET is the total amount of energy expended doing vigorous PA throughout a week. The last three concepts depend on how many days the participant does the activity in a week and how long the participant spends doing these activities on one of those days [22].

The behavior of the participant was classified as sedentary when presenting a score lower than 600 MET/week, as moderate PA when presenting a score ranging between 600 and 3000 MET/week, and as vigorous PA when presenting a score higher than 3000 MET/week [22]. This instrument has an acceptable level of reliability for developed countries with repeatability of around 0.77 (Spearman’s *p*) and a validity criterion of around 0.49 [23,24].

#### 2.2.3. PA Readiness

The Portuguese version of the Physical Activity Readiness Questionnaires for Everyone (PAR-Q+) instrument was used to assess the need for the participant to seek a doctor or a qualified exercise professional before becoming more physically active.

The questionnaire comprises two parts; the first includes seven general health questions and the second includes follow-up questions about the medical condition(s). If the participant answered (1) NO to all the questions in the first part, they were classified as cleared for PA; those who answered YES to one or more were asked to complete the second part. In the second part, if the participant answered (1) NO to all the follow-up questions, they were classified as ready to become more physically active, being advised to consult a qualified exercise professional; those who answered (2) YES to one or more of the follow-up questions were classified as needing further information before becoming more physically active [25]. Each participant was therefore classified as cleared for PA, ready to become more physically active while preferably consulting a qualified professional to help develop a safe and effective PA plan or should seek further information before becoming more physically active or engaging in a fitness appraisal. The PAR-Q+ presents an excellent internal consistency (0.993), an excellent agreement in 93.8% of the questions, and a good to excellent total reproducibility (0.901, 95% CI: 0.887–0.914) [25].

#### 2.2.4. Cardiovascular Risk Stratification

The ACSM’s stratification guidelines were used to assess the risk of atherosclerotic cardiovascular disease (CVD) [2]. The decision tree is divided into three questions, firstly related to the presence or absence of cardiovascular, pulmonary, and/or metabolic disease, secondly about the presence of major signs or symptoms suggestive of cardiovascular, pulmonary, and metabolic disease, and finally the number of risk factors of coronary artery disease (CAD) [2]. The participants were classified as having high, moderate, or low risk of CAD depending on the answers. The participants with major signs or symptoms suggestive of cardiovascular, pulmonary, and/or metabolic disease were classified as high risk. The participants presenting two or more CAD risk factors were classified as moderate risk. The participants presenting less than two CAD risk factors were classified as low risk [2].

### 2.3. Statistical Analysis

Version 28.0 of the Statistical Package for the Social Science (SPSS^®^) software was used for descriptive and inferential statistical analysis, with a level of significance of 0.05. The Kolmogorov–Smirnov test and histogram analysis demonstrated that the data were normally distributed.

The mean, standard deviation, maximum/minimum, and 95% confidence interval values were used for descriptive analysis to characterize the participants’ age, weight, height, body mass index (BMI), and PA level scores. The absolute number and percentage were used for descriptive analysis to characterize the number of participants in terms of history of cardiovascular, respiratory, and/or metabolic disease, family history of coronary disease, high blood pressure, diabetes or prediabetes, high cholesterol, or smoking PA level, PA readiness, and cardiovascular risk stratification.

The Chi-square test was used to compare the history of cardiovascular, respiratory, and/or metabolic disease, family history of coronary disease, high blood pressure, diabetes or prediabetes, high cholesterol, smokers, BMI categories, PA level categories, PA readiness, and cardiovascular risk stratification between academic teachers, non-teacher staff, and students. The one-way ANOVA was used to compare the age, weight, height, BMI, and PA level scores between academic teachers, non-teacher staff, and students.

The sample size was calculated with the StatCalc—Sample Size and Power software, version 7.2, which indicated a total required number of 115 academic teachers, 42 non-teacher staff, and 138 students with a confidence level of 95% and an expected non-respondent rate of 20% [26].

## 3. Results

**Figure 1:**
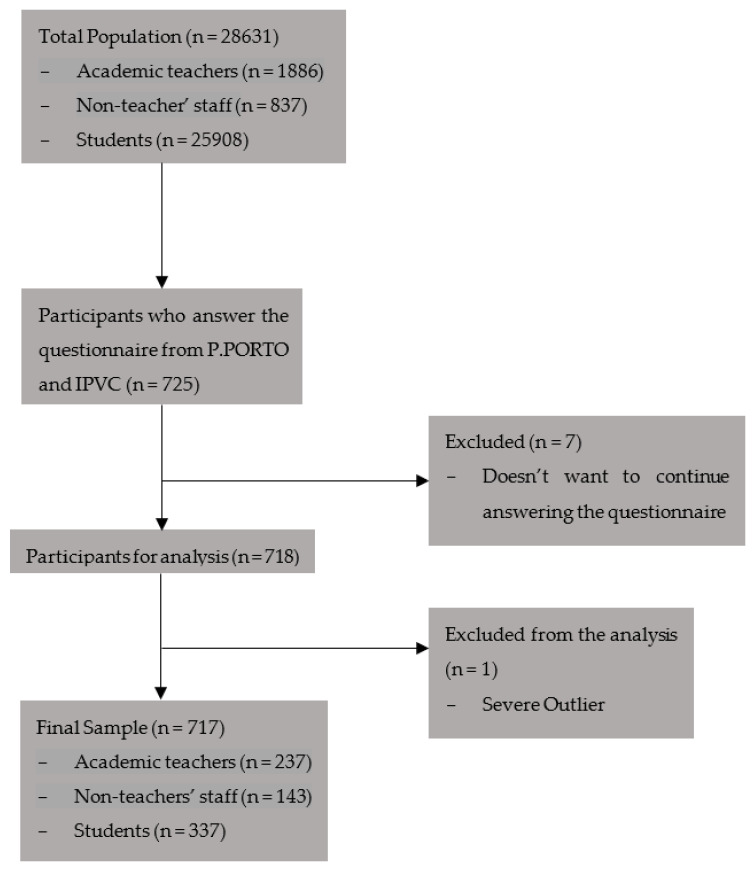
Sample Flow Chart.

A total of 717 participants were included; 237 were academic teachers, 143 were non-teacher staff, and 337 were students (Figure 1).

According to the analysis of Table 1, significant differences were observed between groups in terms of age and BMI. Students are younger, followed by non-teacher staff and academic teachers. The academic teachers presented an increased BMI (40% classified as overweight or superior) compared with non-teacher staff (31% classified as overweight or superior) and students (28% classified as overweight or superior). When comparing the percentage of participants who were underweight, normal weight, and overweight, the results demonstrate that more than 50% of the participants are normal weight. The students are the group with a lower percentage of people overweight and a higher percentage of normal weight.

Regarding health status, 41% of the participants reported health problems, of whom 15.8% were academic teachers, 9.3% were non-teacher staff, and 16.3% were students. Statistical differences between groups were only observed in high cholesterol levels and family history of metabolic, pulmonary, or cardiovascular diseases (Table 1). In both categories, academic teachers had the highest percentage, while students had the lowest percentage. Overall, students pose the least amount of risk, despite the higher rate of history of cardiovascular, respiratory, and/or metabolic disease, along with non-teacher staff, when compared with the group of academic teachers.

### 3.1. PA Level

Overall, 45.75% of the participants presented moderate PA and 36.40% presented vigorous PA, for a total of 82.15% doing any type of PA (Table 2).

On this basis, statistically significant differences in PA levels between groups were found. Specifically, statistically significant differences were found between students and non-teacher staff. Students are more active, with a mean difference between them and non-teacher staff of 990, which is greater than the difference between students and academic teachers, which is approximately 365.

Non-teacher staff show more sedentary behavior and less vigorous activity when compared with academic teachers and students, although non-teacher staff are those who practice more moderate PA.

### 3.2. PA Readiness

According to the values presented in Table 3, over half of the participants are free to engage in significantly greater PA in the academic teachers and students groups, while nearly 30% should seek further information before becoming physically active. In the non-teacher staff group, almost 40% of the participants should seek a health professional and only approximately 35% are cleared for PA. Statistical differences were detected between groups. Academic teachers have higher percentages of participants classified as cleared for PA and lower percentages of participants who should first seek out more information. On the other hand, non-teacher staff had more people that need to seek further information before becoming more physically active and fewer people who were cleared for PA.

### 3.3. Cardiovascular Risk Stratification

Overall, the participants predominantly present a reduced risk of CAD, with 56% of participants at low cardiovascular risk. However, despite the sample’s low risk, according to the values presented in Table 4, 22% of the participants are still classified as high-risk and the remaining 22% as moderate-risk.

Significant differences were observed between groups regarding cardiovascular risk. While students have the highest percentage of people with high cardiovascular risk (26.71%), academic teachers have the lowest percentage of people with high cardiovascular risk (only 13.92%). However, considering the low percentage of students with moderate risk (8.31%) compared to 41% of academic teachers and 23% of non-teacher staff, students also have the greatest percentage of people with low cardiovascular risk (64.98%).

## 4. Discussion

The present research studied the levels of PA and PA readiness, as well as the potential for cardiovascular risk, to determine the need for the development of a MaaS service that promotes sustainable PA moments.

When comparing groups, our data show that students are the least at-risk group. This group presents low levels of sedentarism (14%) and cardiovascular risk (65%), though there is a significant percentage of students with high cardiovascular risk (27%) as well as a significant percentage of people who should seek additional information before becoming more physically active (28%), which must be considered. On the other hand, when comparing academic teachers and non-teacher staff, non-teacher staff seem to have a higher level of risk. Both groups have nearly identical levels of sedentarism: 20% for academic teachers and 23% for non-teacher staff, although academic teachers engage in more vigorous PA (+14%) than non-teacher staff, who engage in more moderate PA (+10%). In terms of cardiovascular risk, academic teachers presented lower levels of risk than non-teacher staff, who showed a higher percentage of people at lower risk (+7%) and a lower percentage of people at higher risk (−11%).

Generally, our findings show that approximately 18% of participants are sedentary, 41% have cardiovascular, respiratory, and/or metabolic health problems, and 44% have moderate or high cardiovascular risk, but despite these findings, only 29% should seek further advice from a health professional before becoming more physically active. Considering the number of people who have health problems, the ACSM guidelines state that before engaging in any sort of PA, individuals should have a health examination and cardiovascular risk stratification to lower the chances of musculoskeletal injury, sudden cardiac death, and myocardial infarction. As a result, the ACSM suggests using a self-guided screening questionnaire for PA and a cardiovascular risk categorization to screen for potential signs, symptoms, and/or risk factors of various cardiovascular, pulmonary, and metabolic diseases [2]. This type of evaluation was considered in this study by first screening for the existence or absence of some cardiovascular, metabolic, or pulmonary diseases, as well as CAD factor risks, and then filling out the PARQ+ questionnaire in the second portion. When compared, the PARQ+ asks about almost all the CVD variables for risk stratification but does not take into consideration the patient’s age, family history of cardiovascular, metabolic, or pulmonary disease, level of obesity, or the user´s PA lifestyle. Thus, the discrepancies in percentages of people with moderate or high cardiovascular risk and the need to consult a health professional before becoming more physically active can be explained by this questionnaire’s lack of information about CAD risks. However, there are more people who say they have cardiovascular, pulmonary, and/or metabolic health diseases than there are people who should obtain additional information before engaging in any type of PA. This can be explained by the likelihood of response bias due to the survey’s length and the fact that the PARQ+ questionnaire was left to the end [27].

When researching the Portuguese university community, it appears that this type of study was only performed previously with students and that it was primarily concerned with their physical conditioning, sedentary lifestyle, and well-being [5,28,29]. In a study conducted in the north of Portugal, 35.7% of the inquired students were sedentary, and they correlated this information with the level of well-being and concluded that students with a high level of sedentary behavior had a lower perception of well-being. When compared with our students, only 14% presented a lower level of PA [5]. Although this research was only applied at the University of Minho [5], in our research, we take into consideration all the schools within two polytechnic institutes. According to the systematic review conducted in 2021, students demonstrated moderate levels of PA, which is consistent with our findings, although they recommend caution due to the scope of their studies and possible disparities in cultural and educational systems [30]. They also discovered disparities in the number of studies that exclusively include students from sports faculties with physical activities as part of the curriculum. As a result, we must take this into account, because P. PORTO and IPVC both have health and sports schools, with classes related to physical training which could boost the levels of PA in our sample [30].

About academic teachers and non-teacher staff, there is not much information. The literature focuses more on academic teachers’ stage of mental health and well-being and does not give any information about the non-teacher staff community [18,31,32]. There is still a study from Poland that analyzed the levels of PA in a community of academic teachers at Pomeranian universities and found low levels of PA. They also attempted to correlate the level of PA to self-efficacy, age, marital status, or the number of children, but found no correlation [33]. When we focus just on academic teachers from our sample, we find that they had higher levels of PA, with 80% practicing moderate or vigorous PA, which opposes the findings of the Polish study. To justify these differences, the same reasons mentioned above for other differences in cultural and educational systems and the differences related to the teaching area can also be applied here.

Looking at the participants’ cardiovascular risk, there is still an important proportion of people with significant cardiovascular risk (44%). A study related to the awareness of cardiovascular risk factors among university students published in 2019 highlights the need for increasing public awareness about the risks of CAD [34]. They showed that CVD awareness among Turkish university students needs to be raised. They agreed that CVD prevention and CAD risk awareness can reduce mortality and morbidity by 80–90%, and that community preventative initiatives should be developed [34,35,36]. As such, the innovative idea of associating the MaaS concept to moments of PA has the same goal of sustaining and growing levels of PA, decreasing levels of sedentarism, and minimizing CAD risks associated with lifestyle to prevent CDV events.

The MaaS concept has the objective of bringing together multiple travel services on one platform to simplify and optimize consumer access, providing a personalized mobility solution linked to individual travel needs [37]. In this way, the project TECH intends to achieve the same thing by delivering a new technology with a holistic perspective by including PA moments into this MaaS solution to comply with the WHO PA recommendations while maintaining a better perspective on community health and their motor function. However, further research in this area is required to determine the effectiveness of this new technology.

### Limitations

We cannot precisely determine what types of areas are represented in the study because polytechnics offer a wide range of teaching fields, including health and sports sciences, technology and management sciences, and educational sciences, among others, and we are unable to distinguish between our participants’ perceived study fields. Another limitation is the lack of participant gender information, which makes it challenging to stratify participants’ cardiovascular risk. The researchers found an approach by considering the lowest age (45 years) for cardiovascular risk classification based on the ACSM guidelines recommendations. Furthermore, to cover all variables in the study, the length of surveys may have resulted in response bias. And lastly, the large number of qualitative variables made it challenging to identify precisely where the true statistical differences were.

## 5. Conclusions

So far as we are aware, this is the first study that assesses PA levels and PA readiness and stratifies cardiovascular risk across the entire university community in Portugal’s north region rather than just in the community of students. The current study found that participants had a level of moderate PA and a decreased risk of CVD, although a considerable percentage of sedentary individuals and with health problems must be considered. Because PA affects not only mental wellness but also the prevention of most cardiovascular, metabolic, and respiratory pathologies, a PA implementation program such as MaaS might be a simple and practical option to increase and sustain the stated behaviors of this group, making it a strategy to consider. Future research on the level of acceptance of this type of PA program’s implementation by the community of Portugal’s north region should be considered to establish the level of usability of this technology by the consumers.

## Figures and Tables

**Table 1:** Mean standard deviation (SD) and 95% confidence interval (CI) regarding age and BMI. Statistical and *p* values obtained from the comparisons between groups were included. The symbol * was added to highlight statistically significant differences.

Study Participants	Academic Teachers (n = 237)		Non-Teacher Staff (n = 147)		Students (n = 337)				
	Mean ± SD (95% CI)	Min–Max	Mean ± SD (95% CI)	Min–Max	Mean ± SD (95% CI)	Min–Max	Z	*p*	
Age (years)	48.28 ± 10.40 (46.94–49.64)	22–72	38.30 ± 14.03 (36.08–40.71)	19–65	26.23 ± 8.63 (25.37–27.15)	19–60	310.418	<0.001 *	P vs. NP < 0.001 * P vs. S < 0.001 * S vs. NP < 0.001 *
Weight (kg)	71.37 ± 15.10 (69.49–73.51)	43–130	66.77 ± 12.94 (64.76–68.91)	43–99	66.51 ± 13.10 (65.03–67.87)	40–112	9.717	<0.001 *	P vs. NP = 0.005 * P vs. S < 0.001 * S vs. NP = 1
Height (cm)	1.70 ± 0.10 (1.68–1.71)	1.42–1.93	1.66 ± 0.09 (1.65–1.68)	1.50–1.92	1.69 ± 0.09 (1.68–1.70)	1.50–1.98	6.461	0.002 *	P vs. NP = 0.001 * P vs. S = 0.570 S vs. NP = 0.023 *
BMI (kg/m^2^)	24.70 ± 4.02 (24.22–25.21)	15.96–43.44	24.13 ± 3.76 (23.54–24.78)	17.48–38.79	23.33 ± 3.90 (22.92–23.74)	15.82–40.98	8.429	<0.001 *	P vs. NP = 0.515 P vs. S < 0.001 * S vs. NP = 0.140
	**n (%)**						**χ2**	***p***	
Underweight (<18.5)	5 (2.1)		4 (2.8)		23 (6.8)		28.190	0.001 *	
Normal weight (18.5–24.9)	136 (57.6)		93 (65.5)		221 (65.6)				
Overweight (25.0–29.9)	68 (28.8)		39 (27.5)		73 (21.7)				
Grade I obesity (30.0–34.9)	24 (10.2)		3 (2.1)		13 (3.9)				
Grade II obesity—severe (35.0–39.9)	2 (0.8)		3 (2.1)		6 (1.8)				
Grade III obesity—morbid (≥40.0)	1 (0.4)		-		1 (0.3)				
	**n (%)**						**χ2**	***p***	
History of cardiovascular, respiratory, and/or metabolic disease	28 (11.8)		18 (12.6)		42 (12.5)		0.657	0.957	
Family history of coronary disease	34 (14.3)		17 (11.9)		30 (8.9)		9.768	0.045 *	
High blood pressure	35 (14.8)		23 (16.1)		31 (9.2)		9.251	0.137	
Diabetes or prediabetes	6 (2.5)		6 (4.2)		8 (2.4)		1.316	0.518	
High cholesterol	55 (23.2)		28 (19.6)		33 (9.8)		23.219	<0.001 *	
Smoker	30 (12.7)		13 (9.1)		33 (9.8)		1.635	0.441	
Total number of participants	237 (33.05)		143 (19.94)		337 (47.00)				

A total of 4 academic teachers, 4 non-teacher staff, and 8 students did not know what cardiovascular, respiratory, or metabolic diseases are. A total of 7 academic teachers, 10 non-teacher staff, and 27 students did not know if they have a family history of coronary disease. A total of 30 academic teachers, 27 non-teacher staff, and 70 students did not know if they have high cholesterol. For BMI and high blood pressure, we used Monte Carlo statistical analysis.

**Table 2:** Mean standard deviation (SD) and 95% confidence interval (CI) of PA levels and percentage of participants in each category. Statistical and *p* values obtained from the comparisons between groups were included. The symbol * was added to highlight statistically significant differences.

Study Participants	Academic Teachers		Non-Teacher Staff		Students				
	Mean ± SD (95% CI)	Min–Max	Mean ± SD (95% CI)	Min–Max	Mean ± SD (95% CI)	Min–Max	Z	*p*	
IPAQ Score	2729.04 ± 2734.76 (2379.07–3079.00)	0–24,556.80	2104.46 ± 1955.07 (1781.27–2427.66)	0–11,880.00	3094.60 ± 2666.21 (2808.91–3380.29)	0–24,840.00	7.542	<0.001 *	P vs. NP = 0.605 P vs. S = 0.279 S vs. NP = <0.001 *
	**n (%)**						**χ2**	***p***	
Sedentary (<600 MET/week)	48 (20.25)		33 (23.08)		47 (13.95)		15.245	0.004 *	
Moderate PA (600–3000 MET/week)	100 (42.19)		75 (52.45)		153 (45.40)				
Vigorous PA (>3000 MET/week)	89 (37.55)		35 (24.48)		137 (40.65)				
Total number of participants	237 (33.05)		143 (19.94)		337 (47.00)				

**Table 3:** Percentage of participants’ PA readiness levels in each category. Statistical and *p* values obtained from the comparisons between groups were included. The symbol * was added to highlight statistically significant differences.

Study Participants	Academic Teachers	Non-Teacher Staff	Students		
	n (%)			χ2	*p*
Cleared for PA	118 (49.79)	49 (34.27)	162 (48.07)	13.589	0.009 *
Ready to become more physically active, preferably after consulting a qualified professional	62 (26.16)	37 (25.87)	79 (23.44)		
Should seek further information before becoming more physically active or engaging in a fitness appraisal	57 (24.05)	57 (39.86)	96 (28.49)		
Total number of participants	237 (33.05)	143 (19.94)	337 (47.00)		

**Table 4:** Percentage of participants cardiovascular risk in each category. Statistical and *p* values obtained from the comparisons between groups were included. The symbol * was added to highlight statistically significant differences.

Study Participants	Academic Teachers	Non-Teacher Staff	Students		
	n (%)			χ2	*p*
Low cardiovascular risk	107 (45.15)	74 (51.75)	219 (64.98)	88.589	<0.001 *
Moderate cardiovascular risk	97 (40.93)	33 (23.08)	28 (8.31)		
High cardiovascular risk	33 (13.92)	36 (25.17)	90 (26.71)		
Total number of participants	237 (33.05)	143 (19.94)	337 (47.00)		

## Data Availability

Data is unavailable due to privacy.
