# Correction: Sousa et al. Physical Activity, Readiness, and Cardiovascular Risk Stratification in the Polytechnics Communities of the Northern Region of Portugal Integrated in Mobility as a Service Concept. *Healthcare* 2023, *11*, 3145

**DOI:** 10.3390/healthcare12100999

**Published:** 2024-05-13

**Authors:** Andreia S. P. Sousa, Diana C. Guedes, José Félix, Soraia Pereira, Rubim Santos

**Affiliations:** Center for Rehabilitation Research (CIR), ESS, Polytechnic of Porto, Rua Dr. António Bernardino de Almeida, 400, 4200-072 Porto, Portugal; dcrg@ess.ipp.pt (D.C.G.); josefelixfelix15@gmail.com (J.F.); sfap@ess.ipp.pt (S.P.); rss@ess.ipp.pt (R.S.)

In the published article [1], there was an error regarding the affiliation. Instead of “Centre for Rehabilitation Research (CIR), School of Health, Polytechnic Institute of Porto, Rua Dr. António Bernardino de Almeida, 400, 4200-072 Porto, Portugal”, the updated affiliation should now include the following: “Center for Rehabilitation Research (CIR), ESS, Polytechnic of Porto, Rua Dr. António Bernardino de Almeida, 400, 4200-072 Porto, Portugal”. The authors state that the scientific conclusions remain unaffected. This correction was approved by an Academic Editor. The original publication has also been updated.
